# Prevalence of HIV infection among non-elderly individuals with hepatitis C in Japan: a population-based cohort study using a health insurance claim data

**DOI:** 10.1186/s12879-022-07152-5

**Published:** 2022-02-21

**Authors:** Kazuhiko Ikeuchi, Kazuya Okushin, Makoto Saito, Eisuke Adachi, Takeya Tsutsumi, Tomoyuki Takura, Hiroshi Yotsuyanagi

**Affiliations:** 1 Department of Infectious Diseases and Applied Immunology, IMSUT Hospital of The Institute of Medical Science, The University of Tokyo, 4-6-1 Shirokanedai, Minato-ku, Tokyo, 108-8639 Japan; 2 Division of Infectious Diseases, Advanced Clinical Research Center, Institute of Medical Science, The University of Tokyo, 4-6-1 Shirokanedai, Minato-ku, Tokyo, 108-8639 Japan; 3 Department of Infection Control and Prevention, Graduate School of Medicine, The University of Tokyo, 7-3-1 Hongo, Bunkyo-ku, Tokyo, 113-8655 Japan; 4 Department of Gastroenterology, Graduate School of Medicine, The University of Tokyo, 7-3-1 Hongo, Bunkyo-ku, Tokyo, 113-8655 Japan; 5 Department of Healthcare Economics and Health Policy, Graduate School of Medicine, The University of Tokyo, 7-3-1 Hongo, Bunkyo-ku, Tokyo, 113-8655 Japan

**Keywords:** Hepatitis C virus (HCV), HIV, Men who have sex with Men (MSM), Injection drug user (IDU), Sexually transmitted infection (STI)

## Abstract

**Background:**

Hepatitis C virus (HCV) has been mainly transmitted through injection drug use, but recently, sexual transmission among men who have sex with men (MSM), which is also a major route of HIV transmission, is increasing. However, the prevalence of HIV and the incidence of other sexually transmitted infections (STIs) among HCV patients have been rarely reported.

**Methods:**

Using a healthcare insurance claim data of employees and their dependents covering seven-million people in Japan, we evaluated HIV prevalence among HCV patients aged 20–59 years. Hemophilia patients were excluded. HIV and HCV were defined by registered diagnoses and receiving viral RNA testing. The time course of HCV and HIV infections was analyzed. Incidences of syphilis, amebiasis, chlamydia, gonorrhea, hepatitis A, and hepatitis B were assessed.

**Results:**

From April 2012 to August 2018, 6,422 HCV patients were identified. HIV prevalence was 0.48% (31/6422, 95% CI [confidence interval]: 0.33–0.68%). HIV was diagnosed after HCV in 3.2% (1/31), before HCV in 58.1% (18/31), and concurrently in 38.7% (12/31). Compared with HCV patients without HIV infection, HCV/HIV co-infected patients were younger (median age, 37 vs 51 years, *p* < 0.001), more likely to be male (30/31 [96.8%] vs 3059/6391 [47.9%], *p* < 0.001), more likely to have other STIs (38.7% [12/31] vs 0.9% [56/6391], *p* < 0.001), and live in Tokyo, the most populous capital city in Japan (67.7% [21/31] vs 11.6% [742/6391], *p* < 0.001). In Tokyo, the HIV prevalence among 20–30 s male with HCV was 18.6% (13/70; 95% CI, 10.3–29.7%).

**Conclusions:**

HIV prevalence among young male HCV patients was very high in Tokyo. HCV/HIV co-infected patients were more likely to acquire HIV before HCV, which is a known feature of MSM. They also had a higher incidence of STIs. These findings suggest that HCV might be prevalent as an STI among MSM particularly in Tokyo.

**Supplementary Information:**

The online version contains supplementary material available at 10.1186/s12879-022-07152-5.

## Introduction

An estimated 1.0% of people in the world are infected with hepatitis C virus (HCV), leading to 475,000 deaths in 2015 [1]. Although HCV is now treatable with direct-acting antivirals (DAAs), it is still a significant public health concern because only around 20% of HCV patients were estimated to be diagnosed and the accessibility of DAAs is still low in low and middle income countries [1].

In patients with chronic HCV infection, concurrent HIV infection is known to be associated with more rapid progression of liver fibrosis, and higher mortality [2–4]. The prevalence of HCV in people living with HIV (PLWH) has been evaluated in many countries, and the global prevalence was estimated to be 2.4% [5]. On the other hand, the prevalence of HIV among patients infected with HCV has been only analyzed in specific populations, such as psychiatric patients [6].

HCV and HIV share similar routes of transmission: injection drug use, contaminated blood products, and sexual contact [7]. In Japan, for example, it is believed that, after the World War II, HCV was spread by illicit methamphetamine due to the post-war confusion [8]. Many hemophilia patients got HIV, HCV, and hepatitis B virus (HBV) due to contaminated blood products, until the 1990s when the screening of these infections became a standard worldwide. Nowadays, injection drug use is regarded as the main mode of transmission of HCV partly because HCV had been considered rarely transmitted by sexual contact; the transmission rate of HCV between non-HIV heterosexual partners were estimated to be one infection per 190,000 sexual contacts [9]. However, in Japan, where there are fewer injection drug users (IDUs) than in the United States and Europe [10], the route of HCV infection in younger people was not well understood.

In the past two decades, however, the sexual transmission of HCV among men who have sex with men (MSM) has become recognized worldwide [11–14].Traumatic sex practice, especially receptive anal intercourse without a condom was reported as a risk for HCV transmission [15]. A characteristic of MSM who are co-infected with HCV/HIV is that they tend to be infected with HIV prior to HCV, implying that HIV is more sexually transmittable and more prevalent than HCV as sexually transmitted infections (STIs) among MSM [7, 16]. Some hypothesized that HIV infection may increase susceptibility to HCV, but recent reports showed that incidence of HCV among non-HIV MSM was comparable to that of HIV-positive MSM [17, 18].

Other STIs are also helpful to assess the sexual activity of the group. For example, syphilis is one of the most common STIs among MSM [19]. Amebiasis and hepatitis A are transmitted by fecal–oral contact and are more common in MSM than in heterosexuals [20, 21]. HBV, chlamydia, and gonorrhea are also seen among heterosexuals, but more common among MSM [22, 23].

Here, we performed a health insurance claim-based cohort study including a general non-elderly adult population across the nation to evaluate the prevalence of HIV among HCV patients. We also analyzed the incidence of STIs among these people to assess their sexual activity.

## Methods

### Study design and description of the data source

We conducted a retrospective analysis using The Tokyo University Health Economy Big Data. This database consisted of medical service data examined by the Social Insurance Medical Fee Payment Fund, a public specialized organization to check health insurance claim of employees and their dependents. Almost all residents in Japan are enrolled in a health insurance (i.e., universal health coverage), and about half are enrolled in one of the employee’s health insurance systems. Our database covers approximately seven million of these people throughout Japan, and approximately five million patients went to hospital at least once between April 2012 and August 2018. Data format was in accordance with the format established by the Japanese Ministry of Health, Labor and Welfare. Using unified IDs, the information on the diagnosis, performed laboratory tests, prescribed medications, and performed medical interventions was able to be followed even if the patient changed hospitals. The authors had access only to patient data with a diagnosis of HCV. The information on the hospitals the patients visited was also available. The following variables were collected from the database: age as of April 2012, sex, the prefecture where the hospital is located (i.e., Tokyo or others). There are 47 prefectures in Japan, and Tokyo is the capital and most populous prefecture. The diagnostic data of HCV, HIV, syphilis, chlamydia, gonorrhea, amebiasis, hepatitis A, hepatitis B, and drug addiction were also collected.

This study was approved by the Research Ethics Committee of the Faculty of Medicine of the University of Tokyo (approval number: 2018167NI), and involved strict data confidentiality in accordance with the Helsinki Declaration and REporting of studies Conducted using Observational Routinely-collected Data (RECORD) Statement.

### Definitions and data preparation

We collected the data on non-elderly adult patients with HCV from this database. Suspected diagnoses were not included. Patients with HCV were defined as those diagnosed with HCV and who received at least one HCV-RNA test during the study period. Most acute and chronic hepatitis C patients were expected to be included by this criterion. Some HCV-seropositive HCV-RNA-negative patients (i.e., spontaneously recovered or previously treated) could have been excluded by this definition.

Because children are rarely infected by injection drug use or sexual contact, and elderly patients often change health insurance after retirement, we excluded patients younger than 20 years and older than 60 years. Hemophilia patients were also excluded because the dominant transmission route of HCV and HIV is contaminated blood products and the prevalence is totally different from the general population [24].

The primary outcome was the prevalence of HIV among the patients with HCV. Patients with HIV were defined as those with the diagnosis of HIV and who received at least one HIV-RNA test during the study period. Prescriptions of antiretroviral drug were also checked for the confirmation of the diagnosis. The secondary outcome was the number of patients who experienced other sexually transmitted infections: syphilis, chlamydia, gonorrhea, amebiasis, hepatitis A, and hepatitis B. Diagnoses of these diseases are shown in Additional file 1: Table S1. The infection episodes of syphilis and amebiasis were defined by their diagnoses and treatment because the treatment is highly specific to each of them. Because antibiotics for chlamydia and gonorrhea are not that specific, they were defined by satisfying all the criteria as follows: diagnoses, receiving nucleic acid amplification tests, and treatment. We counted the number of the patients with HBV diagnosis who received the HBV genotyping test, which is performed only for newly diagnosed hepatitis B. We counted the number of patients who were diagnosed with hepatitis A virus (HAV) and received HAV-IgM test within a month. If these patients were diagnosed with any other hepatitis at the same time (e.g., HBV), they were excluded.

To evaluate the time course of HCV/HIV co-infection, we collected the timing of HIV and HCV diagnoses. When each diagnosis started within 6 months of each other, these infections were defined as being diagnosed at the same time. Even if the diagnosis started before 2012, we could trace back to when the diagnosis started.

### Statistical analysis

This study is a descriptive analysis of a population-based cohort. The prevalence of HIV and an exact 95% confidence interval (CI) was calculated using the binomial distribution. Baseline characteristics and the number of patients infected with each STI were compared between PLWH and non-PLWH. The Mann–Whitney U test was used for continuous variables, and the Fisher’s extract test was used for categorical variables. Statistical significance was defined as 2-sided *p* values of < 0.05.

As a measure of sexual activity, we calculated the incidence of syphilis, which is one of the most common STIs among MSM in Japan. The incidence was compared with a previous report on syphilis incidence among PLWH in Tokyo, of which HCV-seroprevalence was 3% [19]. The observation period here was defined as the length of months on antiretroviral therapy (ART), following this previous study. The 95% confidence interval of the incidence rate of syphilis was calculated based on the Poisson distribution.

## Results

**Fig. 1:**
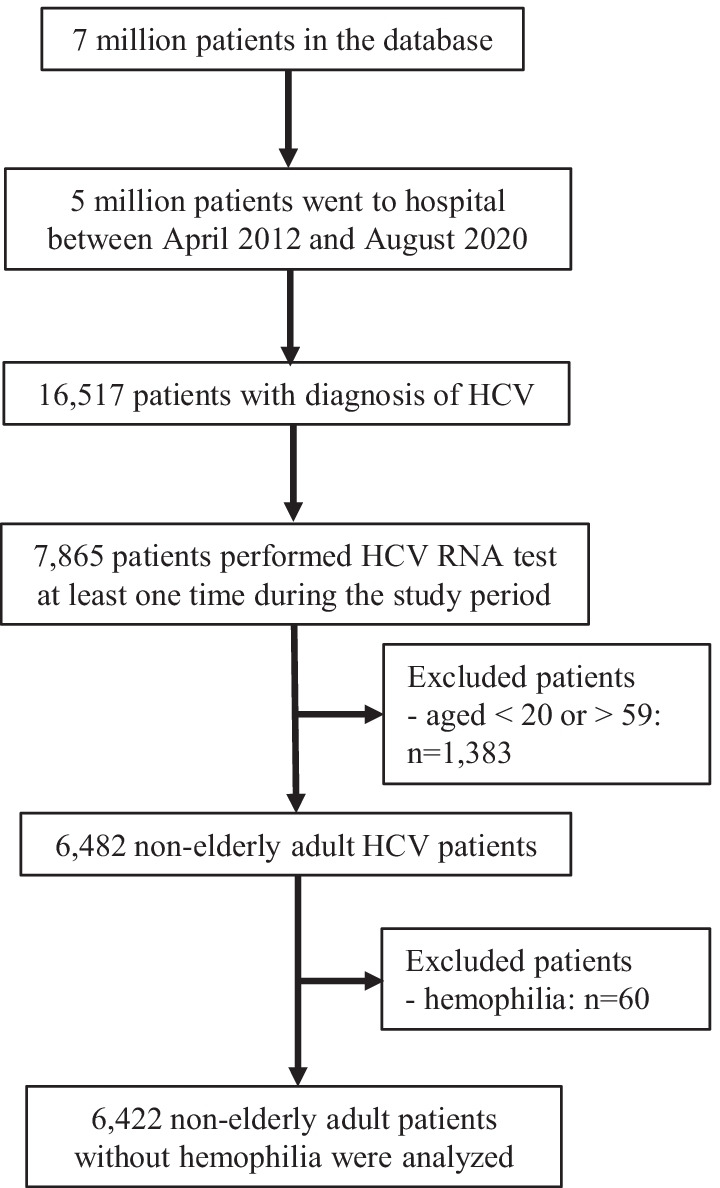
Study flow chart. *HCV* hepatitis C virus

Between April 2012 and August 2018, a total of 16,517 patients with HCV diagnosis were identified (Fig. 1). Among them, 7865 patients were performed at least one HCV-RNA test during the study period, and 1383 patients were excluded by the age criterion. Sixty hemophilia patients were also excluded.

A total of 6422 patients were analyzed. Median age was 51 years (interquartile range [IQR], 44–55 years), and 48.1% (3089/6422) were male. The age distribution of HCV patients was 6.8% (438/6422) in the 20–29, 11.0% (709/6422) in the 30–39, 27.0% (1737/6422) in the 40–49, and 55.1% (3538/6422) in the 50–59 age group, while the decadal age distribution of the whole population in the database was nearly equal across all the age groups. Male was 35.8% (157/438) in the 20–29 s, 39.8% (282/709) in the 30–39, 48.2% (837/1737) in the 40–49, and 51.2% (1813/3538) in the 50–59 age group. In Tokyo, male was 39.0% (23/59) in the 20–29, 44.2% (46/104) in the 30–39, 51.1% (117/229) in the 40–49, and 53.4% (198/371) in the 50–59 age group.

**Fig. 2:**
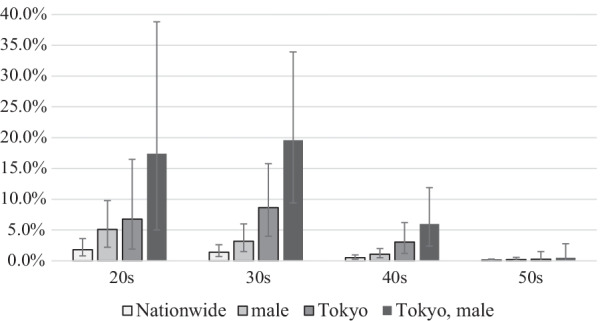
The prevalence of HIV among patient infected with HCV. 95% confidence intervals are shown as whiskers. *HCV* hepatitis C virus

HIV prevalence was 0.48% (31/6422, 95% CI: 0.33–0.68%) among HCV patients. HIV was diagnosed after HCV in 3.2% (1/31), before HCV in 58.1% (18/31), and concurrently in 38.7% (12/31). Characteristics of PLWH and non-PLWH are shown in Table 1. Compared with non-PLWH, PLWH were younger (median age, 37 vs 51 years, *p* < 0.001), more likely to be male (30/31 [96.8%] vs 3,059/6391 [47.9%], *p* < 0.001), and the residents of Tokyo (67.7% [21/31] vs 11.6% [742/6391], *p* < 0.001). Although the number was small, PLWH were more likely to be drug addicts (3.2% [1/31] vs 0.05% [3/6391], *p* = 0.02). In Tokyo, the HIV prevalence among 20–30 s male HCV patients was 18.6% [13/70, 95% CI, 10.3–29.7%] (Fig. 2).

**Table 1:** Characteristics of patients with HCV

Characteristic	PLWH, n = 31	Non-PLWH, n = 6391	*p* value
Age, year*	37 (30–45)	51 (44–55)	< 0.001
Male	30 (96.8%)	3059 (47.9%)	< 0.001
Drug addiction	1 (3.2%)	3 (0.05%)	0.02
Residence in Tokyo	21 (67.7%)	742 (11.6%)	< 0.001
STIs	12 (38.7%)	56 (0.9%)	< 0.001
Treated syphilis	9 (29.0%)	3 (0.05%)	< 0.001
Treated amebiasis	2 (6.5%)	0 (0%)	< 0.001
Treated chlamydia	1 (3.2%)	17 (0.27%)	0.08
Treated gonorrhea	0 (0%)	4 (0.06%)	1.00
HAV -IgM	1 (3.2%)	2 (0.03%)	0.01
HBV -genotyping test	1 (3.2%)	31 (0.49%)	0.14

Data are presented as number (%) unless otherwise indicated

*Median (interquartile range)

*HAV* hepatitis A virus, *HBV* hepatitis B virus, *HCV* hepatitis C virus, *STI* sexually transmitted infection, *PLWH* people living with HIV

Syphilis was the most common STI, and 29.0% (9/31) of PLWH were infected with syphilis during the study period (non-PLWH, 0.05% [3/6391], *p* < 0.001), only one of which was infected with syphilis before HIV diagnosis, and the incidence of syphilis among PLWH was 85.9/1,000 person-years (95% CI, 37.1–169.2). PLWH were more likely to be infected with amebiasis (6.5% [2/31] vs 0% [0/6391], *p* < 0.001), HAV (3.2% [1/31] vs 0.03% [2/6391], *p* = 0.01). There was no statistically significant difference between the number of PLWH and non-PLWH who infected with chlamydia (3.2% [1/31] vs 0.27% [17/6391], *p* = 0.08), gonorrhea (0% [0/31] vs 0.06% [4/6391], *p* = 1.00), and HBV (3.2% [1/31], 0.49% [31/6391], *p* = 0.14). In total, 38.7% (12/31) of PLWH experienced at least one STI, while 0.9% (56/6391) of non-PLWH experienced STI (*p* < 0.001).

## Discussion

We showed that the prevalence of HIV was 0.48% among non-elderly HCV patients in Japan. HCV/HIV co-infected patients were more likely to be young, male, and residents of Tokyo, the capital and most populous prefecture. The prevalence of HIV among 20 s and 30 s HCV male patients in Tokyo were astonishingly high at 18.6%. HCV/HIV co-infected patients had high incidence of STIs and were more likely to acquire HIV before HCV, suggesting that HCV might be prevalent as an STI among MSM particularly in Tokyo.

In Japan, almost all legal residents are under the universal health coverage insurance scheme, provided either by the Employee’s Health Insurance System or the National Health Insurance System. In this study, we analyzed one of the Employee’s Health Insurance Systems, covering about seven-million workers and their dependents. Although the results of laboratory tests are unavailable, we can accurately estimate the number of diseases which have specific laboratory tests or treatment, such as HIV. Since HCV RNA testing is usually performed only on HCV antibody-positive individuals, the health insurance claim database is reliable source of information for estimating the prevalence of HIV among HCV patients.

This is the first report estimating the HIV prevalence among non-elderly HCV patients in Japan, and showed extremely high HIV prevalence in male living in Tokyo. The seroprevalence of HCV was reported as 2.1% in HIV-infected MSM living in Tokyo [25]. In the same report, 21 of 753 PLWH were newly diagnosed with HCV over follow-up period of median 984 (IQR, 539–1557) days, of which only four (19%) were infected by IDU. In addition, Tokyo Metropolitan Infectious Diseases Surveillance Center reported 10 cases of acute hepatitis C in 2018, in which all patients were male, seven were through sexual contact among MSM, one was through injection drug use, one was both, and one was unknown [26]. In this study, HCV/HIV co-infected patients were more likely diagnosed with HIV before HCV, which also supports the idea that HCV is prevalent as an STI among MSM in Tokyo.

In this study, many HCV/HIV co-infected patients were infected with other STIs. Syphilis was the most common STI in the present study, and the incidence rate was 88.3/1000 person-years, which was higher than a previous estimate (43.7/1000 person-years) for HIV-infected MSM living in Tokyo from 2008 to 2015 [19]. Given that one-third (10/31) of the PLWH in our cohort do not live in Tokyo, where the prevalence of syphilis is known to be higher than other areas [19], it is possible that HCV/HIV co-infected PLWH are more sexually active than non-HCV PLWH. In the present study, PLWH were more likely to be infected with amebiasis and HAV, which are usually infected by oral-fecal contact among MSM. In contrast, the number of HBV genotype testing (i.e., the supposedly newly diagnosed HBV infection) among PLWH was not higher than non-PLWH, probably because ART with anti-HBV activity, such as tenofovir or lamivudine, could prevent new HBV infections [27].

There were more female than male HCV patients in younger age even in Tokyo. This can be partly due to differential health-seeking behavior and screening opportunity by sex: males can be underdiagnosed. In general, females are more likely to visit hospital compared with males [28], and are more likely to be diagnosed with HCV earlier than men because of HCV screening in pregnancy. Nonetheless, the HCV transmission route among young female in Japan remains unclear. Among female patients, heterosexual contact is considered not to be the main mode of infection considering the low transmission rate of HCV through heterosexual sex [9]. The proportion of female methamphetamine users is also low in Japan compared with other countries [10]. Tattooing and piercing could be one of the transmission routes [29]. Since HCV antibody testing for pregnant women started in the 1990s, people in their 20 s and 30 s in the early 2010s might have been vertically infected. However, as mentioned above, most acute hepatitis C patients are male in Tokyo recently [26], so the ratio of male to female HCV patients may change in the next few decades.

There were several limitations in our study. Firstly, the diagnosis may not be accurate in some diseases, because the diagnoses are made for health care insurance claim data. For example, in case of window period, HCV-RNA tests could be performed for patients with hepatitis with unknown origin, but this is rare. Therefore, we believe that most of the patients who underwent HCV-RNA test were HCV antibody-positive (i.e., confirmed HCV infection). In the present study, approximately nine thousand patients with HCV diagnosis but without HCV RNA testing were excluded, however, even when they were included, age distribution (data not shown), HIV prevalence (0.41%, 68/16,378), and the order of HCV/HIV diagnoses (HIV was diagnosed after HCV in 1.5% [1/68), before HCV in 45.6% [31/68], and concurrently in 52.9% [36/68]) were almost same. Secondly, PLWH needs regularly visit to hospital for antiretroviral treatment and might have had more opportunities of receiving screening test of HCV and other STIs. Furthermore, it is possible that HIV-induced immunocompromised status made some STIs (e.g., amebiasis [20]) more severe and more detectable. However, even if the prevalence of HCV and incidence of STIs in non-PLWH was underestimated, the very high prevalence of HCV and incidence of STIs in PLWH could not have been overestimated. Thirdly, this database does not cover all the types of health insurance system, and this could cause a selection bias (known as “healthy worker effect”). However, our database mainly covered employed workers and their dependents, so this database is suitable for assessing the prevalence among non-elderly adults. Lastly, we could not follow the data of patients who had changed the types of health care insurance. Therefore, we excluded patients aged > 60 years who were likely to change their insurance after retirement. We assumed the population in the cohort remained stable during the study period.

In conclusion, the prevalence of HIV was 0.48% among patients with HCV in Japan, and 15–20% among non-elderly male in Tokyo. The incidence of STIs was very high among HCV/HIV co-infected patients and were more likely to be diagnosed with HIV prior to HCV. These results might suggest a possibility of the outbreak of HCV among MSM in Tokyo. When non-elderly man living in urban area is diagnosed with HCV, it is necessary to check whether he is already infected with HIV or other STIs. In addition, it may be beneficial to screen PLWH for HCV in areas where an outbreak of HCV infection among MSM is suspected. Healthcare insurance claim data might be useful to uncover the transmission route of HCV and patients at risk of infection.

## Supplementary Information

**Additional file 1. Supplementary Table 1.** Disease definition.

## Data Availability

The datasets generated during the current study are not publicly available due to further uses for clinical studies in the future but are available from the corresponding author on reasonable request.

## Abbreviations

ART: Antiretroviral therapy
CI: Confidence interval
DAA: Direct-acting antiviral
HAV: Hepatitis A virus
HBV: Hepatitis B virus
HCV: Hepatitis C virus
ICD-10: International Classification of Diseases and Related Health Problems, 10th Revision
IDU: Injection drug user
MSM: Men who have sex with men
NAAT: Nucleic acid amplification test
PLWH: People living with HIV
STI: Sexually transmitted infection
