# Supplementary material for: Prevalence of HIV infection among non-elderly individuals with hepatitis C in Japan: a population-based cohort study using a health insurance claim data
Source: BMC Infect Dis. 2022 Feb 21;22:167. doi: 10.1186/s12879-022-07152-5 (PMC8862380; doi:10.1186/s12879-022-07152-5)
Supplement: Supplementary file 1 — Additional file 1. Supplementary Table 1. Disease definition. [file 12879_2022_7152_MOESM1_ESM.docx]

# Title: Prevalence of HIV infection among non-elderly individuals with

Supplementary Table 1. Disease definition

|  | ICD-10 |  | Laboratory testing |  | Treatment |
| --- | --- | --- | --- | --- | --- |
| Hepatitis C | B182 | and | HCV RNA test |  | NA |
| HIV | B20–24 | and | HIV RNA test |  | NA |
| Syphilis | A50–53 |  | NA | and | ≥28-day of oral antimicrobial treatment (i.e., amoxicillin, oral benzylpenicillin, minocycline, and doxycycline) or 14-day of intravenous antimicrobial treatment (i.e., benzathine penicillin G, and ceftriaxone) |
| Chlamydia | A55, 56 | and | NAAT | and | quinolone or macrolide |
| Gonorrhea | A54 | and | NAAT | and | ceftriaxone |
| Amebiasis | A06 |  | NA | and | metronidazole |
| Hepatitis A | A159* | and | HAV IgM test |  | NA |
| Hepatitis B | B181 | and | HBV genotyping test |  | NA |
| Hemophilia | D66 |  | NA |  | NA |
| Drug addiction | T40 |  | NA |  | NA |
| * If patients were diagnosed with other hepatitis at the same time, they were excluded.  Abbreviations: HAV, hepatitis A virus; HBV, hepatitis B virus; HCV, hepatitis C virus; HIV, human immunodeficiency virus; ICD-10, the International Classification of Diseases and Related Health Problems, 10th Revision; NAAT, nucleic acid amplification test | | | | | |
